# Teaching death, spirituality, and palliative care to university students: Novel pedagogical approach

**DOI:** 10.1017/S1478951524001330

**Published:** 2025-01-24

**Authors:** Jeff Clyde G. Corpuz

**Affiliations:** Department of Theology and Religious Education, College of Liberal Arts, Manila, Philippines

**Keywords:** death education, spirituality, palliative care, medical curriculum, experiential learning, holistic patient care, theology, inclusive-pluralism, end-of-life care, interprofessional collaboration

## Abstract

Teaching death, spirituality, and palliative care equips students with critical skills and perspectives for holistic patient care. This interdisciplinary approach fosters empathy, resilience, and personal growth while enhancing competence in end-of-life care. Using experiential methods like simulations and real patient interactions, educators bridge theory and practice. Integrating theological insights and inclusive-pluralism encourages meaningful dialogue, preparing students to address patients’ physical, emotional, and spiritual needs. This holistic pedagogy not only improves patient outcomes but also promotes collaboration and compassion in healthcare.

In contemplating the profound subjects of death, spirituality, and palliative care, one must approach with the clarity and rigor of scientific inquiry, combined with the empathy and understanding inherent in theological and religious/spiritual reflection. These topics, while seemingly disparate, intersect at the core of our human experience and thus merit a cohesive pedagogical approach, especially when engaging the inquisitive minds of college students (Parekh de Campos et al. 2022; Scherg et al. 2021).

Teaching death, spirituality, and palliative care to college students is an essential yet often overlooked component of medical education. A thorough literature review reveals the profound impact that early education on these topics can have on future healthcare professionals. According to a study by Pieters et al. (2019), integrating palliative care education into the medical curriculum significantly enhances students’ competence and confidence in handling end-of-life care situations. This early exposure helps demystify death and dying, allowing students to approach these inevitable aspects of healthcare with greater empathy and understanding. Moreover, incorporating spirituality into the curriculum, as highlighted by Puchalski et al. (2014), can provide students with a holistic approach to patient care, addressing not only physical but also emotional and spiritual needs.

The literature underscores the importance of experiential learning in teaching these sensitive subjects. For instance, a study by Wittenberg-Lyles et al. (2008) emphasizes the benefits of role-playing, simulations, and reflective writing in palliative care education. These methods allow students to engage deeply with the material, fostering a more personal and profound understanding of death and dying. Furthermore, integrating real patient interactions, as suggested by Thyson et al. (2022), can bridge the gap between theoretical knowledge and practical application, enabling students to develop essential communication skills and emotional resilience. Results revealed that contact with real-world patients helps mould the students’ attitude on death and dying. This hands-on experience is crucial in preparing future healthcare professionals to provide compassionate and comprehensive care to patients and their families during the most challenging times.

In addition to enhancing clinical skills, education on death, spirituality, and palliative care can significantly impact students’ personal and professional development. As noted by Sinclair (2011), students who receive comprehensive training in these areas often report a deeper sense of purpose and fulfillment in their work. This training helps them to confront their own fears and misconceptions about death, leading to personal growth and a more profound connection with their patients. Moreover, the literature highlights the positive effects on interprofessional collaboration, as students from various healthcare disciplines learn to work together to provide holistic and patient-centered care. Overall, incorporating education on death, spirituality, and palliative care into the medical curriculum is not only beneficial for students’ professional competence but also for their personal development and well-being, ultimately leading to better patient care (Corpuz 2023c).

Death, the inevitable cessation of biological processes, is often shrouded in fear and misunderstanding (Breitbart 2017a). To teach about death is to confront the finite nature of our existence. College students, standing at the threshold of their adult lives, may view death as a distant abstraction (Bigelow et al. 2022). Yet, instilling a logical understanding of mortality can empower them to live with greater purpose and compassion. From a theological standpoint, death is not merely an end but a transition, a passage to an unknown dimension that has intrigued human consciousness for millennia (Corpuz 2023a). Encouraging students to explore death through the lenses of various religious and philosophical traditions can foster a more comprehensive and respectful dialogue beyond existential guilt and fear of death (Breitbart 2017b).

Spirituality, distinct from organized religion, represents the quest for meaning and connection beyond the material realm (Breitbart 2009). It is a universal human trait, transcending cultural and temporal boundaries. Teaching spirituality involves guiding students to explore their own beliefs, encouraging introspection, and fostering a sense of connectedness with the broader human community and the cosmos. Palliative and supportive care exemplifies the practical application of our understanding of death and spirituality (Corpuz 2023b). This field focuses on alleviating suffering and improving the quality of life for those with serious illnesses. It is where the theoretical meets the practical, where empathy, medical science, and ethical considerations converge (Sabir et al. 2020). Teaching palliative care involves imparting not only medical knowledge but also the values of compassion, dignity, and respect for the individual’s wishes. Students should be encouraged to see patients as whole beings, with physical, emotional, and spiritual dimensions. This holistic approach resonates with the teachings of major religions of the world on the interconnectedness of all things and the importance of viewing problems in their entirety (Corpuz 2024a).

To effectively teach these interconnected subjects, one must adopt an interdisciplinary approach that blends scientific reasoning with ethical and spiritual considerations. Encourage students to question, to seek evidence, and to reflect deeply on their values and beliefs. Provide them with the tools to engage in empathetic communication and ethical decision-making. Incorporating case studies, focus group discussions, personal narratives, and trans- or cross-disciplinary readings can enrich the learning experience, making abstract concepts tangible and relatable. Encourage an environment where students feel safe to express their thoughts and emotions, thus promoting a culture of dialogue and mutual respect. As a theologian, I encourage my students to view contentious issues such as death, afterlife, and salvation through what I call “inclusive-pluralism” which is model that goes beyond exclusivism.

An effective teaching strategy in this context is to find the “common ground” while embracing an diversity through inclusive-pluralism approach. Through the identification of shared human values and universal themes within diverse perspectives on death, spirituality, and palliative care, professors and theologians can create a foundation for mutual understanding and respect in the classroom. This method helps students appreciate the interconnectedness of different beliefs and experiences, fostering a more inclusive and empathetic worldview (Corpuz 2023c). Encouraging open dialogue and respectful debate within a safe space classroom allows students to express their views and learn from one another, promoting a pluralistic approach that values diversity while seeking unity in common human experiences.

In teaching topics such as death, spirituality, and palliative care, educators are not merely imparting knowledge but are fostering the growth of compassionate, reflective, and holistically developed individuals. However, a significant number of individuals remain unaware of the importance of palliative and supportive care. Corpuz (2024b) encouraged the immediate access to palliative and supportive care in the Philippines. This holistic perspective equips students to face the complexities of life with wisdom, courage, and humanity, embodying the very principles we seek to instill.

A novel pedagogical approach to teaching death, spirituality, and palliative care in medical education holds immense promise in transforming how future healthcare professionals perceive and handle end-of-life care. Furthermore, incorporating spirituality into the curriculum enriches students’ understanding of patient care, encouraging them to address not only the physical but also the emotional and spiritual needs of their patients. This holistic perspective is crucial in developing a well-rounded healthcare professional capable of delivering comprehensive care. The literature consistently highlights the positive outcomes of such education, including personal growth, a deeper sense of purpose, and improved interprofessional and multisectoral collaboration.

In conclusion, university students need to understand and embrace these fundamental aspects of human existence as we prepare them not only for their professional futures but also for the profound personal journeys they will undertake. Adopting innovative teaching strategies in the context of death, spirituality, and palliative care is critical for preparing medical students to navigate the complexities of end-of-life care with empathy and competence. This novel pedagogical approach promises to enhance both the professional and personal development of future healthcare providers, ultimately leading to improved patient outcomes and a more compassionate healthcare system.

## Supporting information

Corpuz supplementary material Corpuz supplementary material

## Supplementary material

The supplementary material for this article can be found at https://doi.org/10.1017/S1478951524001330.
