# Supplementary material for: Teaching death, spirituality, and palliative care to university students: Novel pedagogical approach
Source: Palliat Support Care. 2025 Jan 24;23:e36. doi: 10.1017/S1478951524001330 (PMC13166390; doi:10.1017/S1478951524001330)
Supplement: Corpuz supplementary material [file S1478951524001330sup001.docx]

**Teaching death, spirituality, and palliative care to university students:**

**Novel pedagogical approach**

Jeff Clyde G. Corpuz, PhD

Department of Theology and Religious Education, College of Liberal Arts,

2401, De La Salle University, 1004 Manila, Philippines

**Corresponding author:**

Jeff Clyde G. Corpuz, PhD

Department of Theology and Religious Education, College of Liberal Arts,

2401, De La Salle University, 1004 Manila, Philippines

E-mail: [jeff.corpuz@dlsu.edu.ph](mailto:jeff.corpuz@dlsu.edu.ph)

<https://orcid.org/0000-0003-1517-4866>
